# Shexiang Baoxin Pill (MUSKARDIA) reduces major adverse cardiovascular events in women with stable coronary artery disease: A subgroup analysis of a phase IV randomized clinical trial

**DOI:** 10.3389/fcvm.2022.1002400

**Published:** 2022-10-21

**Authors:** Haiming Shi, Jingmin Zhou, Changsheng Ma, Fusui Ji, Yang Wu, Yulan Zhao, Jun Qian, Xiaolong Wang

**Affiliations:** ^1^Department of Cardiology, Huashan Hospital, Fudan University, Shanghai, China; ^2^Department of Cardiology, Zhongshan Hospital, Fudan University, Shanghai, China; ^3^Department of Cardiology, Beijing Anzhen Hospital, Capital Medical University, Beijing, China; ^4^Department of Cardiology, Beijing Hospital of the Ministry of Health, Beijing, China; ^5^Department of Cardiology, Dongfang Hospital Affiliated to Beijing University of Chinese Medicine, Beijing, China; ^6^Department of Cardiology, The Second Affiliated Hospital of Zhengzhou University, Zhengzhou, China; ^7^Department of Cardiology, The Center Hospital of Ma’anshan, Ma’anshan, China; ^8^Department of Cardiovascular, Shuguang Hospital, Shanghai University of Traditional Chinese Medicine, Shanghai, China

**Keywords:** MUSKARDIA, women, stable coronary artery disease, angina, major adverse cardiovascular event

## Abstract

**Background:**

A previous phase IV trial revealed sex as a potential effect modifier of MUSKARDIA efficacy in stable coronary artery disease (CAD).

**Objective:**

To assess the clinical effect of MUSKARDIA as a supplemental treatment to optimal medical therapy (OMT) in stable CAD cases.

**Methods:**

This study was a subgroup analysis of a multicenter, randomized, double-blinded, placebo-controlled phase IV clinical study. Eligible individuals underwent randomization to the oral MUSKARDIA and placebo groups and were treated for 24 months. All participants received OMT according to existing guidelines. The primary composite outcome was the major adverse cardiovascular event (MACE), included cardiovascular death, non-fatal myocardial infarction (MI), or non-fatal stroke. The secondary composite outcome encompassed all-cause mortality, non-fatal MI, non-fatal stroke, hospitalization for unstable angina and/or heart failure, and undergoing coronary procedure/surgery during treatment. Safety signals, especially cardiovascular adverse events (AEs), were analyzed.

**Results:**

The female subgroup included 776 participants (384 and 392 in the MUSKARDIA and placebo groups, respectively). The occurrence of the primary composite outcome was lower in the MUSKARDIA group compared with placebo-treated individuals (HR = 0.27, 95%CI: 0.09–0.83; *P* = 0.02), but the secondary composite outcome showed no significant difference (HR = 0.77, 95%CI: 0.47–1.25; *P* = 0.29). The MUSKARDIA group had reduced incidence of cardiovascular AEs compared with placebo-treated cases (2.9% vs. 5.6%).

**Conclusion:**

As a supplemental treatment to OMT, 24-month administration of MUSKARDIA is effective and safe in female stable CAD cases.

**Clinical trial registration:**

[https://clinicaltrials.gov/], identifier [NCT01897805].

## Introduction

Coronary artery disease (CAD) is characterized by atherosclerotic coronary artery narrowing, which mostly shows no early symptoms but could result in stable or unstable angina and/or myocardial infarction (MI) with increased thickening or plaque rupture of the wall of the coronary arteries (1, 2). Commonly reported risk factors for CAD are dyslipidemia, tobacco smoking, hypertension, a family history of CAD, diabetes, and obesity. Complications are acute coronary syndrome (ACS), ST-elevation myocardial infarction (STEMI), acute heart failure, arrhythmia, and sudden-death. Ischemic heart disease caused by CAD is the largest cause of death, with 12.7% of all worldwide deaths (3).

Aspirin, β-blockers, lipid-lowering agents, and angiotensin-converting enzyme inhibitors (ACEIs) are recommended as optimal drugs for the secondary prevention of coronary heart disease (1, 2, 4, 5). The combination of aspirin and a statin is routinely administered as a secondary preventive measure to decrease the risk of cardiovascular events in stable CAD (6–9). Frequently, this approach is inadequate, and a residual CAD risk still exists in many patients (10–12).

Currently, CAD is considered as a major life-threatening disease for the Chinese population. Furthermore, many Chinese CAD patients, especially women, do not tolerate aspirin due to gastrointestinal reactions, aggravated pulmonary disease, and hyperuricemia, among others (13–15). Traditional Chinese medicine (TCM) is a possible supplemental therapy that has been applied for a long time for treating CAD (16, 17). Shexiang Baoxin Pill (MUSKARDIA), utilized for CAD and angina for over 40 years, comprises seven materia medica or extracts: Moschus, Radix Ginseng, Calculus Bovis, Cortex Cinnamomi, Styrax, Venenum Bufonis, and Borneolum Syntheticum. The bioactive substances in MUSKARDIA are muscone, ginsenosides, storax, bufadienolides, cinnamic acid, arenobufagin, and borneol (18–21). A phase IV trial revealed that MUSKARDIA, as a supplement to optimal medical therapy (OMT), is safe and decreases the frequency of angina in patients with stable CAD, with a trend toward reducing the occurrence of major adverse cardiovascular events (MACEs) (22). These beneficial effects could be related to MUSKARDIA-associated coronary artery dilation and coronary angiogenesis, which increase the coronary blood flow (23). In TCM, MUSKARDIA is an aromatic drug for yang-activation, used to treat chest impediment (CAD).

Coronary artery disease is a major cause of death in women. CAD prevalence is lower in women than in men, but there are significant differences in epidemiology, risk factors, pathophysiology, clinical manifestations, treatment, and patient prognosis (24–28). In a previous phase IV trial (22), univariable and multivariable analyses showed that sex is independently associated with the clinical outcomes of MUSKARDIA. Therefore, it is of great significance to improve the treatment of women with CAD and ameliorate their prognosis. Therefore, further analysis was carried out on the female subgroup to confirm the efficacy of MUSKARDIA in the female CAD population.

## Methods

### Study design

This was a subgroup analysis of the randomized, double-blinded, placebo-controlled, phase IV MUSKARDIA trial performed in 97 clinical centers in China (22), after approval from the ethics committees of various participating centers. Each patient provided signed informed consent prior to any study procedure.

### Study population

The key inclusion criteria of the trial included age ≥ 18 years, stable myocardial ischemia symptoms for at least 1-month, acute MI course > 6 months, percutaneous coronary intervention (PCI) or coronary artery bypass graft (CABG) administered > 6 months ago, at least one major coronary artery with ≥50% stenosis, and a negative urinary pregnancy test. The key exclusion criteria were CABG or PCI scheduled during the trial, serious cardiovascular diseases, serious pulmonary disorders, diabetes with poor glycemic control, poor blood pressure control despite hypertension treatment, serious liver or kidney diseases, or, if non-menopausal, refusal to use proper contraceptive methods. In this subgroup analysis, female participants in the full analysis set (FAS) were selected.

### Randomization and blinding

In the trial, eligible patients were randomized 1:1 to receive MUSKARDIA or placebo. Central randomization with blocks of four and no stratification was used for generating codes with numbers for each treatment group. The placebo was provided by Shanghai Hutchison Pharmaceuticals. Participants, investigators, and central study staff were blinded to grouping.

### Intervention

Eligible and consenting cases were enrolled in a 28-day run-in period, when they were administered standard treatment for stable CAD based on current guidelines. The participants were next administered oral MUSKARDIA (2 pills t.i.d., 135 mg totally) or placebo (2 pills t.i.d., 135 mg totally). The study drug was administered for 24 months consecutively or until discontinuation because of an adverse event (AE). Both groups received OMT according to clinical guidelines during the study.

### Outcomes and assessments

The primary composite efficacy outcome was the MACE, included cardiovascular death, non-fatal MI, and non-fatal stroke. The secondary composite outcome encompassed all-cause death, non-fatal MI, non-fatal stroke, hospital admission for unstable angina or heart failure, and PCI or CABG during the study. The Seattle Angina Questionnaire (SAQ) was used to evaluate angina symptoms. Safety outcomes included the types and frequencies of AEs. This subgroup analysis specifically focused on cardiovascular AEs.

### Statistical analysis

SAS 9.2 (SAS Institute, USA) was performed for data analysis. Continuous variables with an approximately normal distribution are mean ± standard deviation (SD), and were compared between groups by the *t*-test. Continuous data with skewed distribution were expressed by median and interquartile range (IQR), and compared between groups by the Mann–Whitney *U*-test. Categorical data were expressed by *n* (%), and compared by the Fisher’s exact test or the chi-square test. Cumulative incidence curves were generated by the Kaplan–Meier (K–M) method for outcomes. The log-rank test and Cox’s proportional hazards model were used to compare clinical outcomes between the two groups.

## Results

### Study population

The randomized phase IV MUSKARDIA trial was performed from July 2011 to August 2015 with 2,674 patients, of which female participants were included in this subgroup analysis. Therefore, 776 participants were analyzed, including 384 and 392 in the MUSKARDIA and placebo groups, respectively.

The participants were 65.6 ± 8.8 and 65.9 ± 8.5 years old in the MUSKARDIA and placebo groups, respectively, and body mass index (BMI) values were 24.1 ± 3.2 and 24.2 ± 3.2 kg/m^2^, respectively (*P* > 0.05 for both age and BMI). The overall aspirin and statin use rates were 96.6 and 91.4% in women administered MUSKARDIA, respectively, versus 96.4 and 93.9% in placebo treated cases, respectively. In addition, 49.5 and 53.6% of patients in the MUSKARDIA and placebo groups were under angiotensin receptor blocker (ARB)/ACEI treatment at enrollment, respectively. Table 1 shows the detailed baseline characteristics of the female patients analyzed.

**TABLE 1:** Baseline information of the female subgroup.

	MUSKARDIA (*n* = 384)	Placebo (*n* = 392)	*P*
Age (years)	65.6 ± 8.8	65.9 ± 8.5	0.54
<65 years	172 (44.8%)	167 (42.6%)	0.54
≥65 years	212 (55.2%)	225 (57.4%)	
BMI (kg/m^2^)	24.1 ± 3.2	24.2 ± 3.2	0.57
Smoking	57 (14.8%)	54 (13.8%)	0.85
**Medical history**			
Hypertension	230 (59.9%)	237 (60.5%)	0.87
Chronic kidney disease	134 (34.9%)	151 (38.5%)	0.30
Diabetes	106 (27.6%)	112 (28.6%)	0.76
**Baseline medication**			
Aspirin	371 (96.6%)	378 (96.4%)	0.89
Statins	351 (91.4%)	368 (93.9%)	0.19
ARB/ACEI	190 (49.5%)	210 (53.6%)	0.25

BMI, body mass index; ARB, angiotensin receptor blocker; ACEI, angiotensin-converting enzyme inhibitor. Data are *n* (%) or mean ± SD.

### Efficacy outcomes

**FIGURE 1:**
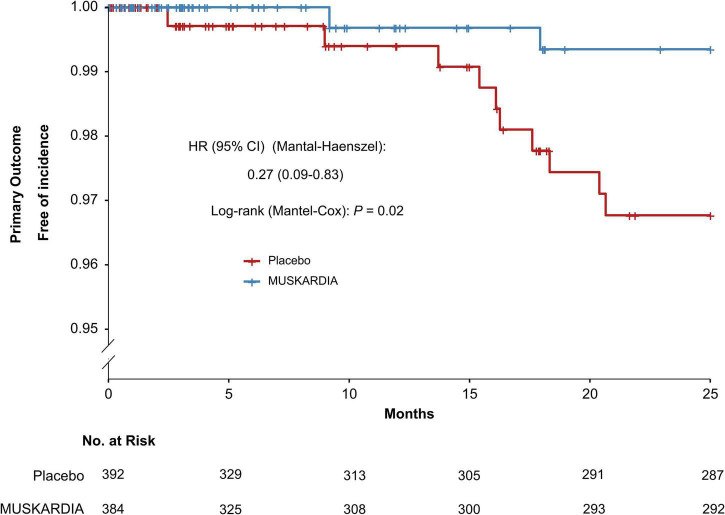
Kaplan–Meier curve analysis of the primary composite outcome. Hazard ratio = 0.27 (95% confidence interval: 0.09–0.83), *P* = 0.02.

**FIGURE 2:**
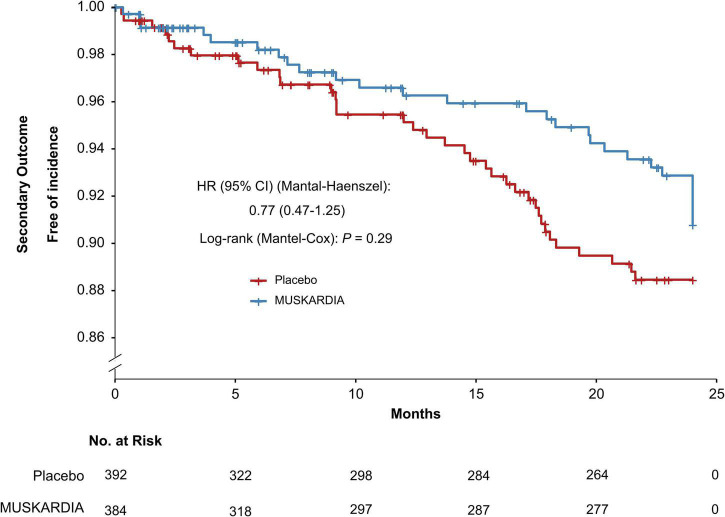
Kaplan–Meier curve analysis of the secondary composite outcome. Hazard ratio = 0.77 (95% confidence interval: 0.47–1.25), *P* = 0.29.

The incidence rates of the primary composite outcome were 0.5% (*n* = 2) and 2.6% (*n* = 10) in the MUSKARDIA and placebo groups at 24 months, respectively (Table 2). From Month 18, Kaplan–Meier curves for the two groups became separated, with significantly reduced MACE occurrence in MUSKARDIA treated cases in comparison with the placebo group (HR = 0.27, 95%CI: 0.09–0.83; *P* = 0.02) (Figure 1). The incidence of the secondary composite outcome was 7.3% (*n* = 28) in the MUSKARDIA group versus 9.2% (*n* = 36) in the placebo group at 24 months (HR = 0.77, 95%CI: 0.47–1.25; *P* = 0.29) (Table 2 and Figure 2). Among them, non-fatal MI was significantly reduced in MUSKARDIA group (0.3% vs. 2.0%, *P* = 0.04).

**TABLE 2:** Efficacy outcomes.

Outcome	MUSKARDIA (*n* = 384)	Placebo (*n* = 392)	*P*
**Primary composite outcome**	2 (0.5%)	10 (2.6%)	0.02
Cardiovascular death	0	1 (0.3%)	>0.99
Non-fatal myocardial infarction	1 (0.3%)	8 (2.0%)	0.04
Non-fatal stroke	1 (0.3%)	1 (0.3%)	>0.99
**Secondary composite outcome**	28 (7.3%)	36 (9.2%)	0.36
All-cause death	3 (0.8%)	2 (0.5%)	0.68
Non-fatal myocardial infarction	1 (0.3%)	8 (2.0%)	0.04
Non-fatal stroke	1 (0.3%)	1 (0.3%)	>0.99
Hospitalization for unstable angina	22 (5.7%)	27 (6.9%)	0.56
Hospitalization for heart failure	2 (0.5%)	1 (0.3%)	0.62
Received coronary angioplasty	5 (1.3%)	8 (2.0%)	0.58

Data are *n* (%).

Other outcomes, including all-cause mortality (0.8 and 0.5% in the MUSKARDIA and placebo groups, respectively), and non-fatal stroke (0.3 and 0.3%, respectively) were similar between the two groups (Table 2). The results of the SAQ showed that all dimensions were significantly improved during the treatment in both groups except for physical limitations. Both groups had comparable data (Table 3).

**TABLE 3:** Seattle Angina Questionnaire score during the treatment.

Seattle Angina Questionnaire	Group/months	0	1	3	6	9	12	18	24
Physical limitation	MUSKARDIA	78.9 ± 7.0	78.7 ± 7.1	78.7 ± 7.3	79.1 ± 5.5	79.4 ± 4.5	79.1 ± 5.8	79.3 ± 5.5	78.6 ± 6.7
	Placebo	77.9 ± 8.3	77.8 ± 9.0	78.0 ± 8.0	78.6 ± 6.5	79.1 ± 4.7	78.8 ± 5.6	79.2 ± 6.6	78.4 ± 9.7
Angina stability	MUSKARDIA	59.8 ± 23.3	66.1 ± 21.0*	68.3 ± 22.4*	69.0 ± 23.5*	68.0 ± 23.5*	69.9 ± 24.0*	70.2 ± 24.8*	71.4 ± 23.9*
	Placebo	61.4 ± 22.7	66.1 ± 20.7*	70.1 ± 22.4*	70.5 ± 23.5*	70.2 ± 23.6*	71.9 ± 23.0*	75.6 ± 21.9*	73.7 ± 22.9*
Angina frequency	MUSKARDIA	79.9 ± 19.4	84.3 ± 17.6	86.5 ± 16.2*	87.8 ± 15.2*	87.8 ± 15.8*	87.7 ± 16.3*	88.3 ± 16.2*	89.2 ± 15.3*
	Placebo	79.9 ± 19.1	83.4 ± 18.7	86.6 ± 16.7*	86.9 ± 16.3*	88.4 ± 14.9*	89.7 ± 14.4*	89.2 ± 14.4*	89.0 ± 14.0*
Satisfaction with treatment	MUSKARDIA	72.0 ± 15.3	72.3 ± 14.9	72.8 ± 14.7	74.4 ± 13.5	74.3 ± 12.9	74.9 ± 13.0	76.0 ± 12.6*	76.3 ± 13.7*
	Placebo	71.0 ± 15.4	72.2 ± 14.8	73.5 ± 14.6	74.3 ± 13.5	75.4 ± 13.5	76.5 ± 12.8*	77.3 ± 13.4*	76.5 ± 12.9*
Cognition of disease	MUSKARDIA	57.1 ± 21.7	59.5 ± 20.5	61.1 ± 19.7	63.1 ± 17.9*	63.6 ± 16.9*	63.2 ± 17.7*	65.2 ± 17.3*	66.4 ± 17.0*
	Placebo	58.2 ± 20.0	59.8 ± 20.3	61.3 ± 19.0	63.5 ± 18.8*	65.3 ± 18.3*	66.3 ± 17.6*	68.5 ± 18.3*	67.6 ± 18.0*

*P < 0.05 vs. baseline.

### Safety outcomes

The types, frequencies, and severities of AEs were compared between the two groups (Table 4). The numbers of participants with at least one AE were 55 (14.3%) and 78 (19.9%) in the MUSKARDIA and placebo groups, respectively. Totally 9 (2.3%) and 12 (3.1%) participants in the MUSKARDIA and placebo groups had at least one SAE, respectively. The occurrence of at least one cardiovascular AEs was 2.9% (*n* = 11) and 5.6% (*n* = 22) in the MUSKARDIA and placebo groups, respectively (Table 4).

**TABLE 4:** Adverse events (AEs) and cardiovascular AEs.

AE	MUSKARDIA (*N* = 384)	Placebo (*N* = 392)
At least one AE	55 (14.3%)	78 (19.9%)
At least one SAE	9 (2.3%)	12 (3.1%)
Had at least one cardiovascular AE	11 (2.9%)	22 (5.6%)
Stable angina	3 (0.8%)	4 (1.0%)
Unstable angina	2 (0.5%)	4 (1.0%)
Palpitation	1 (0.3%)	4 (1.0%)
Coronary artery disease	0	3 (0.8%)
Chronic heart failure	1 (0.3%)	0
Premature ventricular contraction	1 (0.3%)	1 (0.3%)
Acute myocardial infarction	0	1 (0.3%)
Atrial fibrillation	1 (0.3%)	1 (0.3%)
Atrial tachycardia	0	1 (0.3%)
Heart failure	1 (0.3%)	0
Acute coronary syndrome	0	1 (0.3%)
Others	3 (0.8%)	2 (0.5%)
Abnormal liver enzyme	3 (0.8%)	5 (1.3%)
Discomfort in liver area	1 (0.3%)	0
Urinary tract infection	3 (0.8%)	3 (0.8%)
Abnormal urine test	5 (1.3%)	7 (1.8%)
Abnormal kidney function	4 (1.0%)	7 (1.8%)

AE, adverse event; SAE, serious adverse event. Data are *n* (%).

## Discussion

This study aimed to assess the effect of MUSKARDIA as a supplement to OMT in women with stable CAD. The results suggested that 24-month treatment with MUSKARDIA is effective and safe in female cases as an add-on to OMT for CAD. The parent trial showed that in the total population of male and female participants, add-on of MUSKARDIA to OMT for 24 months in patients with stable CAD is safe and significantly reduces angina rate and angina stability at 18 months (22). There was also a trend toward decreased MACEs after MUSKARDIA treatment.

The results of the present subgroup analysis are not exactly consistent with those of the parent trial (22). In the parent study, MUSKARDIA reduced MACE occurrence by 26.9% after 2 years of treatment, but the statistical difference was not significant. Nevertheless, in this *post-hoc* analysis, MUSKARDIA showed significant benefit in reducing MACE risk in women with stable CAD (HR = 0.27, 95%CI: 0.09–0.83; *P* = 0.02). Of course, the parent trial included both males and females, and differences in CAD characteristics between sexes (24–28) could have led to some dilution of the effects of MUSKARDIA in the entire study population. Indeed, compared with men, women have more non-obstructive coronary lesions, of which coronary microvascular disease represents a major pathogenetic mechanism of CAD in females, with different occurrence rates in plaque stability and rupture risk, coronary artery spasm, and spontaneous coronary artery dissection (SCAD) (29–31). In addition, the incidence of stroke (which is a part of MACEs) is elevated in females compared with males (32, 33), and the prevalence of angina is also elevated in women (34). Because sex hormones protect women against CAD until menopause (24–28), symptomatic CAD occurs at an older age in women compared with men. Finally, CAD management is more aggressive in men than in women (35–37), which could also play a role here. Of note, because of the small number of events, MUSKARDIA had a significant effect only when considering MACEs as a single endpoint, and the assessment of individual MACEs did not yield significant differences. Additional large studies are necessary to address this issue. Still, females have significant differences from males in terms of CAD epidemiology, risk factors, pathophysiology, clinical manifestations, treatment, and prognosis (24–28), highlighting the need for studies specifically examining women. In this subgroup analysis, the primary efficacy outcome was reduced by MUSKARDIA in the female subgroup (0.5%) compared with the parent trial (1.9%). There were also fewer at least one AEs in the MUSKARDIA group (14.3%) compared with the placebo (19.9%) among female participants. Besides, 17.7% patients (*n* = 236) were treated with MUSKARDIA had at least one AE in the parent trial. Therefore, MUSKARDIA appears to exert a protective effect in women with stable CAD by decreasing the residual risk after OMT.

Kaplan–Meier curve analysis revealed an overt separation starting at 18 months, suggesting MUSKARDIA was superior to the placebo in terms of efficacy, consistent with the parent trial that showed a difference in angina occurrence starting at 18 months of treatment (22). It is generally believed that TCM has favorable effects on CAD over long-term administration (16).

The SAQ is a questionnaire specifically designed to examine angina symptoms. It is one of the most widespread questionnaires that assess angina-specific health status, quantitating angina symptoms and determining their impacts on the quality of life (38). In the present study, all participants received OMT, leading to a low occurrence of CAD events, and MUSKARDIA supplementation further decreased the occurrence rates of MACEs. Since all participants received OMT (including aspirin and statin), the major risk factors for CAD and MACEs were controlled, and MUSKARDIA could reduce the residual CAD risk. The lack of difference in SAQ results between the two groups could be due to a lack of sensitivity of the SAQ to quantify such residual risk. Additional studies are therefore warranted to address the above issue.

Safety, especially after prolonged treatment, represents an important issue with TCM application (39). This study addressed such problem with a large-scale trial over 2 years that included women with CAD. Participants in the MUSKARDIA group showed lower occurrence rates for all types of AEs compared with the placebo group, especially cardiovascular AEs. These safety data support prolonged MUSKARDIA application in female CAD cases. However, further trials are warranted to assess the long-term AEs of TCM. The major AEs in the MUSKARDIA group were cardiovascular AEs, stable angina, and atrial fibrillation, most of which were grade 1 or 2, indicating that patients had high MUSKARDIA tolerability.

In addition, in the original trial, all included females had negative urine pregnancy test results and used proper contraception throughout the study (if non-menopausal). Currently, the safety of MUSKARDIA for the fetus has not been demonstrated, and pregnant women are not recommended to take MUSKARDIA.

The current analysis had multiple limitations. First, despite its pre-specification, this study was a subgroup analysis. The strength of the results might be lower than that of the original trial. Second, the relatively small sample size of this sub-group analysis, based on gender, might increase the possibility of Type II statistical error. Third, further data that could help explain MUSKARDIA’s effects in female patients with stable CAD were not collected. Forth, some important information such as menopause and coronary artery lesions was not recorded. Further studies need to be conducted to confirm the current observations.

In conclusion, MUSKARDIA is effective and safe for residual CAD risk in women with stable CAD administered OMT, and can be recommended for long-term use in female patients with stable CAD.

## Data availability statement

The original contributions presented in the study are included in the article/supplementary material, further inquiries can be directed to the corresponding author.

## Ethics statement

The protocol was approved by the ethics committee at each participating center. The patients/participants provided their written informed consent to participate in this study.

## Author contributions

HS carried out study conception and design, data analysis, and manuscript drafting. All authors organized the database, revised the manuscript, and approved the final version of the manuscript.
